# Molecular characterization of Hepatitis C virus 3a in Peshawar

**DOI:** 10.1186/s12879-016-1488-0

**Published:** 2016-04-18

**Authors:** Amina Gul, Nabeela Zahid, Jawad Ahmed, Fazli Zahir, Imtiaz Ali Khan, Ijaz Ali

**Affiliations:** Institute of Basic Medical Sciences, Khyber Medical University Peshawar, Peshawar, Pakistan; Department of Biosciences, COMSATS Institute of Information Technology Islamabad, Islamabad, Pakistan; IBGE, The University of Agriculture Peshawar, Peshawar, Pakistan; Department of Entomology, The University of Agriculture Peshawar, Peshawar, Pakistan

**Keywords:** Hepatitis C, Peshawar KP, HCV 3a, Pakistan

## Abstract

**Background:**

The purpose of this study was to explore molecular epidemiology of HCV genotype 3a in Peshawar based on sequencing and phylogenetic analysis of *Core* region of HCV genome.

**Methods:**

Chronically infected Hepatitis C virus infected patients enrolled under the Prime Minister Hepatitis C control program at three Tertiary care units of Peshawar [Khyber Teaching Hospital Peshawar, Lady Reading Hospital Peshawar, Hayat Abad Medical Complex Peshawar] were included in this cross sectional observational study. Qualitative detection of HCV and HCV genotyping was carried out by a modified reverse transcription-polymerase chain reaction (RT-PCR) and type specific genotyping assay. The *Core* gene of HCV genotype 3a was amplified, cloned and sequenced. The sequences obtained were used for phylogenetic analysis using MEGA 6 software.

**Results:**

Among the 422 (82.75 %) PCR positive samples, 192 (45.5 %) were identified as having HCV genotype 3a infection. HCV *Core* gene sequencing was carried out randomly for the characterization of HCV 3a. Nucleotide sequence analysis of the obtained viral genomic sequences based on partial HCV 3a *Core* gene sequences with reference sequences from different countries showed that our sequences clustered with some local and regional sequences with high bootstrap values.

**Conclusion:**

HCV 3a is highly prevalent in Peshawar, Pakistan and its phylogenetics based on *Core* gene sequences indicate the prevalence of different lineages of HCV 3a in Peshawar which may have consequences for disease management strategies causing more economic pressure on the impoverished population due to possible antiviral resistance.

## Background

Hepatitis C virus (HCV), first identified in 1989 is the primary cause of chronic liver diseases including cirrhosis (60–85 %) and hepatocellular carcinoma [1], with an annual mortality rate of 3.5-5.5 million due to complications of end stage liver diseases [2]. An estimated 185 million people (3 % of the world’s population) are infected worldwide with relatively high prevalence rates in developing countries [3, 4]. In Pakistan, approximately 10–17 million people are chronically infected with HCV [5, 6] with an overall prevalence rate of 5 % [7]. Frequency of HCV infection differs among the four provinces with an estimated 1.1 million people infected in KPK [8, 9].

HCV is an enveloped, positive sense ssRNA genome [10] in the family Flaviviridae consisting of an oral reading frame (ORF) approximately of 9.6 kb in length [11]. HCV genome shows substantial nucleotide sequence variability in both the structural and nonstructural coding regions, with different isolates of HCV showing as much as 30 % nucleotide sequence divergence over the entire genome which is sufficient to alter the antigenic and biological characteristics of seven major genotypes of HCV [12]. These genotypes have distinct geographical distributions. Although HCV genotypes 1, 2 and 3 appear to have a worldwide distribution, their relative prevalence varies from one geographic area to another [13]. Identification of HCV genotype is extremely important as different genotypes are relevant to the epidemiology and clinical management of chronic HCV infection and is the strongest predictive parameter for sustained virological response [14].

The primary means to identify and classify new genotypes is either by phylogenetic analysis of sequences or by measures of pairwise sequence similarity [15]. Although it is generally true that longer sequences are more informative for classification, it is usually possible to identify genotypes by sequence comparison of relatively short sub genomic regions of HCV [16]. While region encoding the 5′ non-coding region (5′ NCR) is too conserved for HCV subtypes identification, sequencing and phylogenetic analysis of nucleotide sequences amplified in the region of the genome encoding the *Core* protein is frequently used for classification of HCV into genotypes and subtypes [17–19]. HCV *Core* gene has sufficient genetic diversity and can produce topologically identical trees to those obtained upon analysis of complete genome sequences [20].

Molecular epidemiological studies previously conducted in Peshawar, Pakistan have indicated that the most prevalent HCV subtype is 3a accounting for 70–90 % of HCV infections based solely on type specific PCR based genotyping methods developed 15 to 20 years ago [21, 22]. At present, there is little information about the genetic history and evolution of HCV genotype 3a in Peshawar which needs to be investigated for several reasons including increasing antiviral resistance in the case of HCV 3a in Peshawar, KP province and its evolutionary relationship with other regional or global isolates. As the prevalence of HCV 3a has been reported to be very high among the general population of Peshawar, therefore, we embarked on the current study to investigate it at molecular level by Type-specific assay, sequencing of the *Core* gene and phylogenetic analysis in order to have a clear epidemiological picture of the prevalent HCV 3a isolates.

## Methods

### Sampling

Chronically infected Hepatitis C virus infected patients enrolled under the Prime minister Hepatitis C control program at three Tertiary care units [Khyber Teaching Hospital Peshawar, Lady Reading Hospital Peshawar, Hayat Abad Medical Complex Peshawar] willingly participated in this observational study. A non-probability convenient sampling technique was used to collect data. Written consent was obtained from all the eligible study participants for participation and publication of the data and the Institutional Ethics Committee (Khyber Medical University Ethics Board) approved the study. Blood samples were collected from the patients in sterile vacutainers and sera extraction was carried out at the Institute of Biotechnology and Genetic Engineering Peshawar.

### RNA Extraction

Total RNA was extracted from each sample using FavorGen RNA isolation Kit (Favorgen Biotech corp, Taiwan, CAT No FAVNK 001) according to the manufacturer’s instructions. The isolated RNA was stored either stored at-80C or immediately used for RT PCR.

### HCV Genotyping

Reverse Transcription PCR followed by Type-specific PCR for identification of HCV subtypes was carried out essentially according to [23] with modifications in the Universal and Type-specific primers according to the latest HCV sequencing data (Table 1).

**Table 1:** Modifications in the Universal and Type specific primers

Primers	Sequences
UOS^a^	GTGCCCC GGGAGGTCTGT
UIS^b^	GTAGACCGTGCATCATGAGCAC
UOAS^c^	ATGTACCCCATGAGATCG GC
HCV^d^_3a	CGCTCCGACGCGCCTTGG
HCV_3b	CGCTCGGAAGTCTTACGTAC
HCV_1a	GGGATAGGCTGACGTCTACCT
HCV_1b	TGTCGCCTTCCACGAGGTTG
HCV_2a	CCACGTGGCTGGGACCGC
HCV_2b	TGGGGCCCCAAGTAGGACGA

^a^Universal Outer Sense

^b^Universal Inner Sense

^*c*^Universal Outer Anti Sense

^d^Hepatitis C Virus

### Amplification of HCV 3a *Core* gene

A representative number of samples (58/192), which turned out positive for HCV 3a by the Type-specific assay described earlier, were used for a second round of RNA extraction, RT PCR and subsequent regular nested PCR for the amplification of *Core* gene using gene-specific primer [24].

### Cloning and sequencing of the *Core* gene

The *Core* gene products were eluted from agarose gel using Pure Link™ Quick Gel Extraction Kit (https://www.thermofisher.com/pk/en/home.html). The eluted products were cloned in pGEMT-Easy vector and transformed in DH5-alfa strain of E.coli (Promega). Sequencing PCR of the *Core* gene was carried out using vector-specific sense and antisense primers in a sequencing PCR (Big Dye Deoxy Terminator method). Bidirectional sequencing run was performed using ABI PRISM 310 DNA sequencer [Applied Biosystems].

### Blast analysis

In case of each isolate, three independent clones were sequenced. The respective sequences were aligned and consensus sequences were derived. The sequences were blasted using NCBI Blast tool and genotype authentication was further validated with the help of an online software HCV Genotyper (http://www.bioafrica.net/rega-genotype/html/subtypinghcv.html). The confirmed and curetted sequences representing the diversity of 58 sequenced isolates were submitted to GenBank

### Phylogenetic analysis

HCV *Core* gene sequences from various geographical regions of the world were retrieved from the online sequence repositories and Phylogenetic tree was constructed by using Mega6 [25].

### Statistical analysis

SPSS version 20 was used for data analysis. The qualitative variables were described using percentage and the quantitative variables were described using the mean, median and standard deviation.

## Results

**Fig. 1:**
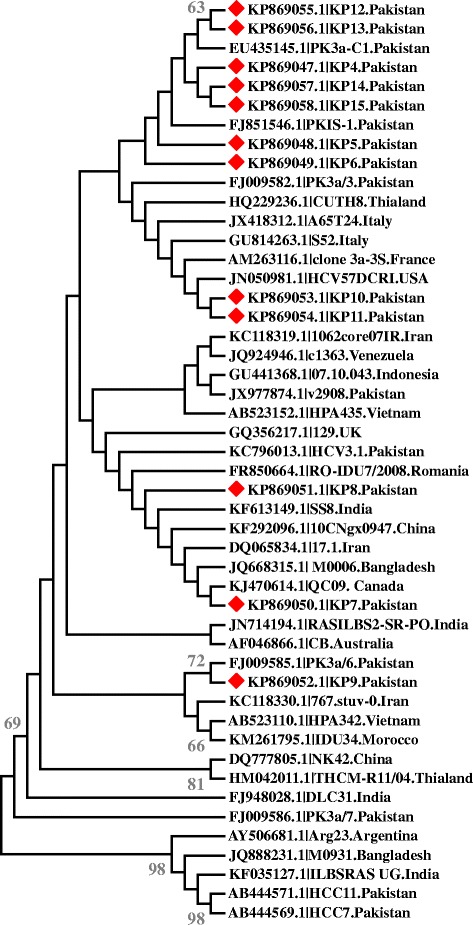
Phylogenetic tree of HCV 3a *Core* gene sequences constructed by Maximum Likelihood algorithm (MEGA 6 Software) with Bootstrap values shown on the branches. Tree shows the phylogenetic relationship of twelve newly reported sequences, marked in red with 36 *Core* gene sequences from different geographical regions. The isolate/country/Accession no of the sequences are shown in figure

For identification of HCV genotype 3a infection among the actively infected HCV samples, RT PCR followed by Type specific nested PCR based genotyping assay was carried out. Genotyping analysis revealed that out of the 422 (82.75 %) samples processed. Out of 422 actively infected patients, 257 (61 %) were females and 165 (39 %) were male) HCV genotype 3a was identified as the most abundant HCV subtype 192 (45.50 %). Mean age of patients included in the study was 38 ± 11.8 (Mean ± SD. For authentication of HCV genotype 3a, *Core* gene amplification and sequencing was carried out by randomly selecting a representative number (58/192) of samples and it was established that the patients had HCV 3a infection. These results were also validated by an online sub-typing tool (http://www.bioafrica.net/rega-genotype/html/subtypinghcv.html). Detailed sequence and phylogenetic analysis of the obtained viral genomic sequences was carried out. Phylogenetic tree was developed with Maximum Likelihood algorithm (1000 bootstrap replicates). To analyze HCV genotype 3a of Peshawar origin, the available subtype 3a sequences of the *Core* gene were retrieved from NCBI and aligned with the respective subtype identified in this study. Pair wise nucleotide and deduced amino acid sequence comparison of our sequences with 36 reported sequences from different countries was performed which indicated that some of the HCV 3a isolates of Peshawar clustered together with each other and with isolates from Pakistan (KP869047, KP869048, KP869049, KP869055, KP869056, KP869057, KP869058), while others clustered closer to European, Indian, Iranian or Chinese HCV sequences with high bootstrap support (Fig. 1). Two of the representative isolates (KP869053 and KP869054) were clustered with the European isolates. Isolate KP869050 and KP869051 shared homology with both the Indian, Iranian and Chinese isolates. Moreover isolate KP869052 was found closely related to those from Iran and Pakistan.

## Discussion

Accumulation of nucleotide substitutions in the HCV genome results in diversification and evolution into seven major genotypes and a series of subtypes [26]. The classification of HCV by viral genotype is not only important for appropriate treatment regimen and assessment of global viral evolution but their epidemiological data can reveal transmission activity and migration movement of infected individuals from the endemic area [27–29]. These viral types and subtypes show differing distribution in different geographic regions which have provided investigators with an epidemiologic marker that can be used to trace the source of HCV infection in a given population [30]. Sequencing and phylogenetic analysis of HCV *Core* gene nucleotide sequences has earlier been used for identification and classification of HCV isolates into genotypes and subtypes [19]. HCV genotype 3a is the most abundant in Pakistan and it has earlier been documented that HCV genotype 3a is relatively more responsive to therapy as compared to genotype 1 or 4 [28, 29, 31]. However, in Pakistan resistance is being observed in case of relatively responsive and easy to treat HCV genotype 3a [32].

The present study is the first report describing the genotypic and evolutionary analysis of HCV 3a isolated from chronic HCV infected patients of Peshawar, based on a modified genotyping assay and subsequent sequencing and phylogenetic analysis. Investigation of the different circulating genotypes and their evolution is not only crucial for epidemiological and clinical analysis but might be helpful for the improvement of diagnostic tests and treatment regimens [33]. HCV infection is highly prevalent in Pakistan with an overall prevalence rate of 5 % [34]. The estimated HCV prevalence in KP province is 1.1 % [8]. In this study, out of total 510 seroactive patients investigated, 82.75 % were actively infected with HCV having high proportion of HCV genotype 3a (45.50 %) which is in agreement with most recent studies in Peshawar and Pakistan reporting HCV 3a to be the most prevalent genotype [35, 36], however the percentage prevalence is lower than the previous reports claiming much higher prevalence of HCV genotype 3a (60–74 %) [22, 37]. One possible explanation for this changing HCV genotype 3a landscape could be the change in epidemiological pattern over times as a result of people migration [38] or the inadequate sensitivity of old genotyping assays to correctly type the circulating viral strains due to substantial genetic heterogeneity inherent to RNA viruses [26]. Moreover the emerging resistance to interferon therapy experienced in case of HCV genotype 3a [32] might possibly be due to the less prevalent genotypes other than HCV 3a or variants of HCV 3a evolved overtimes which can cause substantial economic and health burden over the infected population. Internationally accepted guidelines for the treatment of hepatitis C are rarely followed in KPK and people undergo successive therapies once the initial response of antiviral drugs is negative [32]. Due to lack of awareness, the practice mentioned above is not only causing economic and health related losses but is also contributing towards the evolution of more resistant HCV 3a types.

Molecular evolutionary analysis of the obtained viral genome sequences revealed that some HCV 3a isolates of Peshawar clustered closer to local isolates (Fig. 1) indicating the previous existence of similar types in other parts of Pakistan, which may have spread to Peshawar, KPK province via various transmission routes. Some of the HCV 3a isolates grouped with European, south Asian, Iranian and Chinese HCV 3a isolates (Fig. 1). Peshawar city is located in the northwestern region of Pakistan. It shares international border with the Afghanistan which has been home to conflicts for the past 40 years resulting in migration of various ethnic groups into and out of Afghanistan. These migrations have changed epidemiological patterns of various pathogens overtimes including HCV. Phylogenetic analysis indicate that foreign presence in Afghanistan and migration of Afghanis to Peshawar might have contributed towards the spread of isolates which are genetically closer to European, Indian, Chinese and Iranian isolates as HCV genotype 3a is also highly prevalent in neighboring countries including China [38], India [39] and Iran [40].

This study has some limitations. Due to convenient sampling, selection bias might have occurred. Moreover we have characterized HCV 3a entirely on the bases of partial core gene sequences. To elucidate the epidemiology of emerging HCV 3a in Peshawar and to further improve the accuracy of diagnostic assays and treatment regimens, there is a need to analyze complete coding sequences of more diverse regions of HCV genome on a comparatively large scale.

## Conclusion

This study concludes that HCV 3a is highly prevalent in Peshawar, Pakistan and its phylogenetics based on *Core* gene sequences indicate the prevalence of different lineages of HCV 3a in Peshawar which may have consequences for disease management strategies causing more economic pressure on the impoverished population due to possible antiviral resistance.

### Availability of data and materials

The datasets supporting the conclusions of this article are available in the GenBank [National Center for Biotechnology Information] repository under the following accession numbers [KP869047, KP869048, KP869049, KP869050, KP869051, KP869052, KP869053, KP869054, KP869055, KP869056, KP869057, KP869058]

## Abbreviations

HCV: hepatitis C virus
KP: khyber pakhtunkhwa
